# Identification of Early Zygotic Genes in the Yellow Fever Mosquito *Aedes aegypti* and Discovery of a Motif Involved in Early Zygotic Genome Activation

**DOI:** 10.1371/journal.pone.0033933

**Published:** 2012-03-23

**Authors:** James K. Biedler, Wanqi Hu, Hongseok Tae, Zhijian Tu

**Affiliations:** 1 Department of Biochemistry, Virginia Tech, Blacksburg, Virginia, United States of America; 2 Virginia Bioinformatics Institute, Virginia Tech, Blacksburg, Virginia, United States of America; University of Leuven, Belgium

## Abstract

During early embryogenesis the zygotic genome is transcriptionally silent and all mRNAs present are of maternal origin. The maternal-zygotic transition marks the time over which embryogenesis changes its dependence from maternal RNAs to zygotically transcribed RNAs. Here we present the first systematic investigation of early zygotic genes (EZGs) in a mosquito species and focus on genes involved in the onset of transcription during 2–4 hr. We used transcriptome sequencing to identify the “pure” (without maternal expression) EZGs by analyzing transcripts from four embryonic time ranges of 0–2, 2–4, 4–8, and 8–12 hr, which includes the time of cellular blastoderm formation and up to the start of gastrulation. Blast of 16,789 annotated transcripts vs. the transcriptome reads revealed evidence for 63 (P<0.001) and 143 (P<0.05) nonmaternally derived transcripts having a significant increase in expression at 2–4 hr. One third of the 63 EZG transcripts do not have predicted introns compared to 10% of all *Ae. aegypti* genes. We have confirmed by RT-PCR that zygotic transcription starts as early as 2–3 hours. A degenerate motif VBRGGTA was found to be overrepresented in the upstream sequences of the identified EZGs using a motif identification software called SCOPE. We find evidence for homology between this motif and the TAGteam motif found in Drosophila that has been implicated in EZG activation. A 38 bp sequence in the proximal upstream sequence of a kinesin light chain EZG (*KLC2.1*) contains two copies of the mosquito motif. This sequence was shown to support EZG transcription by luciferase reporter assays performed on injected early embryos, and confers early zygotic activity to a heterologous promoter from a divergent mosquito species. The results of these studies are consistent with the model of early zygotic genome activation via transcriptional activators, similar to what has been found recently in Drosophila.

## Introduction

The maternal-zygotic transition is the period in embryogenesis when maternal RNAs and proteins are degraded and activation of the zygotic genome occurs with nascent RNA transcription [1]. It is established for species of divergent taxonomic groups that there are two waves of zygotic transcription, a minor wave occurring early where a subset of zygotic genes is activated, and a major wave occurring later when widespread zygotic transcription occurs [2], [3]. In Drosophila the minor wave starts at mitotic cycle 8 and occurs during the pre-cellular blastoderm period. Several genes transcribed during the minor wave are involved in sex determination, patterning, and cellularization [3], [4]. The major wave occurs at mitotic cycle 14, which consists of widespread zygotic transcription and is when cellularization of the blastoderm takes place [2], [4]. Large-scale studies have been performed to identify the zygotic genes of Drosophila [5], [6]. In contrast, virtually nothing is known about EZGs in mosquitoes. The first major groundwork for early embryonic development in mosquitoes was published over 3 decades ago [7]. Based on that work, the first and second mitotic divisions occur between 1 and 2 hrs. Nuclei start to migrate to the periphery by the end of 4 hr and complete migration by 6 hr, forming a syncytial blastoderm. Cellularization of the blastoderm initiates at 8 hr and is completed by 10 hr. In comparison to *Ae. aegypti*, Drosophila early development is much faster with initiation of the syncytial blastoderm and initiation of blastoderm cellularization occurring at 1.5 hr and about 2.5 hr, respectively [8], [9].

Historical models for generalized transcriptional silencing mechanisms of the zygotic genome involve chromatin-mediated repression, transcriptional machinery deficiency, and transcriptional repression or abortion by rapid cell cycles [1]. Specific transcriptional activators involved in early zygotic genome activation have remained elusive until recently. In mouse, a maternal protein BRG1 was found to play a role in zygotic genome activation via alteration of histone methylation [10]. In Drosophila, a heptamer CAGGTAG motif was first recognized in the upstream sequences of some sex determination genes [11], [12]. Later this motif was shown to not only be associated with sex determination genes, but was more widely present in the upstream sequences of genes transcribed during the pre-cellular blastoderm period, before widespread zygotic transcription commences [4], [6]. In addition, the CAGGTAG motif was found to be a member of a group of variant motifs named the TAGteam, all of which are overrepresented in genes expressed pre-cellular blastoderm in Drosophila [4]. Later, a transcription factor named Zelda was identified as one factor that binds the TAGteam motif and was shown to be a key activator of the Drosophila early zygotic genome [13]. The discovery of the TAGteam motif and Zelda in Drosophila demonstrated that EZG activation (pre-cellular blastoderm) required positively-acting transcription factors, complementing historical models of zygotic genome activation.

One purpose of this research was to study the activation and expression of EZGs, as relatively little is known in mosquitoes as previous studies have focused on expression more broadly over development [14], [15]. We previously described the kinesin light chain EZGs that were discovered during the initial part of this study [16]. Here we present the first systematic whole-transcriptome genome-wide investigation of EZGs in a mosquito species. While EZGs may be defined as those genes being transcribed during the pre-cellular blastoderm period (up to 8 hr in *Ae. aegypti*), we focus mostly on those involved at the onset of transcription during 2–4 hr. Furthermore, we only consider those genes which do not have any maternally-derived transcripts. Using high-throughput transcriptome sequencing (RNA-Seq) combined with bioinformatic methods, we have covered time points from 0 to 12 hr, up to the start of gastrulation. Before this work, cis-regulatory elements involved in zygotic genome activation in mosquitoes had not been found. We have identified a motif that is at least partially responsible for the activation of EZGs and find support for homology between this motif and the Drosophila TAGteam motif.

Another purpose of this study was to identify EZGs that may contribute promoters for zygotic-specific expression of antidote genes for a mosquito maternal-effect dominant embryonic arrest (*Medea*) gene-drive system, as has been engineered in Drosophila [17]. Engineered *Medea* is comprised of two basic components as part of the same transgenic cassette, a maternally-expressed toxin for which all eggs are exposed, and a zygotically-expressed antidote that will rescue those offspring which inherit the *Medea* cassette. The maternal toxin can be a miRNA that targets the 5′ untranslated region (5′UTR) of a maternal-effect transcript that is critical for embryonic development. The zygotically-expressed antidote from the *Medea* cassette that encodes for the same product as the targeted maternal-effect gene has a unique 5′UTR that is insensitive to the toxin. Therefore, offspring from a heterozygous *Medea*-carrying female will not survive unless they have inherited at least one allele of the *Medea* cassette from either parent. Thus *Medea* can spread relatively quickly in a population [17]. Effective disease management may be achieved if we can develop *Medea* in mosquitoes and use it to drive pathogen-resistant genes into mosquito populations. In this study, we have identified several candidate genes that may facilitate development of promoters for early zygotic-specific expression of transgenic antidotes for a *Medea* gene-drive system.

## Results

### Sequencing of the *Ae. aegypti* early zygotic transcriptome

**Figure 1:**
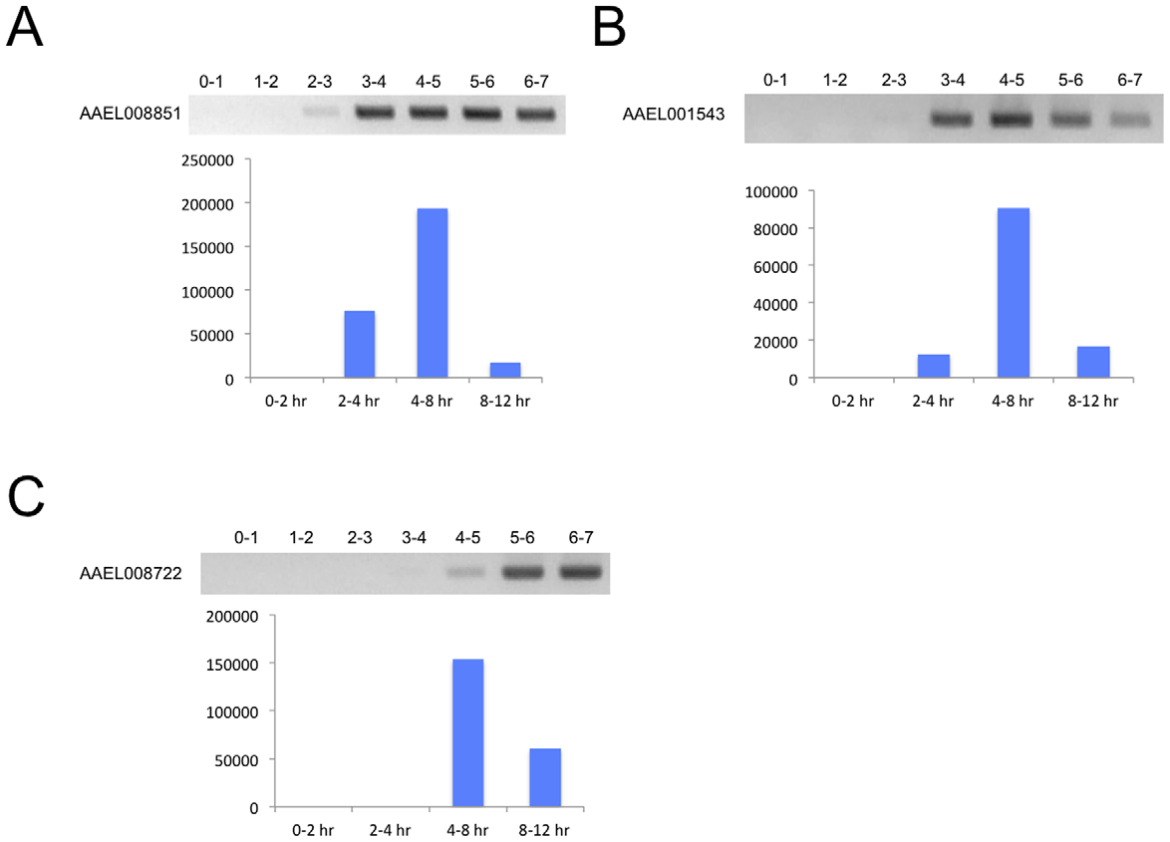
RT-PCR validation of transcriptome data. A) AAEL008851, an example of an EZG gene where both RT-PCR and transcriptome sequencing show expression at the 2–3 hr time range. B) AAEL001543, a zygotic gene with a similar profile as AAEL008851 but which has less expression in the 2–4 hr time range (note very faint band from RT-PCR at 2–3 hr was present that may not be discernable in image). C) AAEL008722, a zygotic gene having expression detected later at 4–5 hr by PCR. Normalized expression values of each transcript from the transcriptome data are at the bottom of each figure. Note that AAEL008722 is not included in our “EZG set” as it is not present until the 4–8 hr time range in the transcriptome data.

Transcriptome sequencing of four embryonic time points (0–2, 2–4, 4–8, and 8–12 hr) was performed and described previously [16] (see Materials and Methods). Data has been submitted to the Gene Expression Omnibus with accession GSE34480. For a summary of the transcriptome sequencing results, see Additional File 1 from [16]. To help validate the transcriptome data and investigate potential candidates for our EZG promoter search, we performed RT-PCR with RNA isolated from embryos from 0–7 hr in one-hour intervals using 25 amplification cycles. Figure 1 shows some of our results demonstrating different expression profiles that are consistent with the transcriptome data (see [16] for a profile of a transient EZG *KLC2.1*, or AAEL011410, the promoter of which is a major focus in this manuscript). Expected product sizes amplified in Figure 1A), B), and C) are 561, 517, and 716 bp, respectively. No introns are annotated for AAEL008851 or AAEL001543 in VectorBase but AAEL008722 has a predicted 243 base intron. However, the RT-PCR product size for AAEL008722 indicates an absence of splicing and the predicted intron does not have stop codons and is in-frame relative to the adjacent predicted exons. In addition, hits are present in the transcriptome database across this sequence with similar frequency as in the predicted coding sequence.

### Defining an early zygotic gene set with statistical significance

To identify the “pure” EZG set from the transcriptome data, we first applied two filtering criteria: transcripts could not be present in the 0–2 hr time range and must be present at least in the 2–4 hr time range. Presence at 0–2 hr indicates a maternal transcript as zygotic transcription is not expected at this time (see Materials and Methods). To be inclusive, the minimum requirement for evidence of transcript presence was one read hit detected by Blast. After applying these filters, 1,529 candidate transcripts were identified, some being alternative transcripts from the same gene and some being paralogous transcripts with high shared nucleotide identities (see File S1). These may not be distinguished from each other by our method. DEGseq [18] was then used to determine which of the 1,529 candidate transcripts had significant expression at 2–4 hr, resulting in 63 (P<0.001) and 143 (p<0.05) transcripts. Many or most of the 1,529 transcripts could be the result of background expression. To be stringent, in our analyses below we focus on the 63 transcripts (61 genes) meeting the p-value<0.001 filter (examples in Figure 1, see File S1 for DEGseq output).

### *Ae. aegypti* early zygotic gene structure

**Figure 2:**
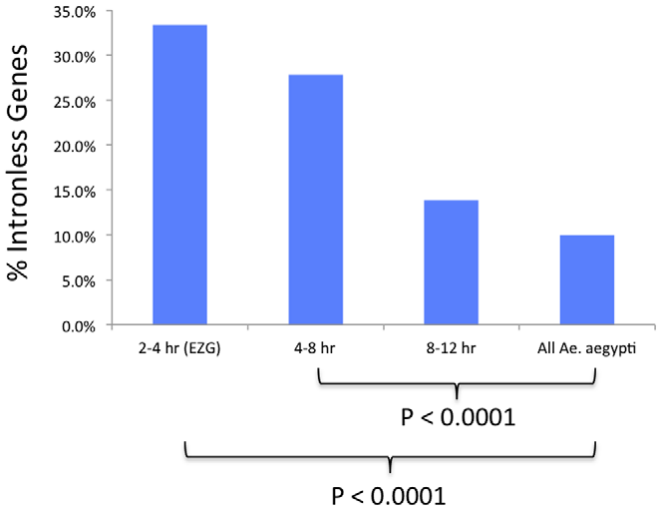
*Ae. aegypti* EZGs have more intronless genes. The percentage of intronless genes was compared between EZGs, 4–8 hr, 8–12 hr and all *Ae. aegypti* genes. P-values calculated by the Chi-squared test are shown for two comparisons. See File S2 for numbers of genes with and without introns for each group. See File S3 for transcript IDs for the 2–4, 4–8, and 8–12 hr groups.

In Drosophila it was reported that activation of the zygotic genome starts with the transcription of about 59 genes at mitotic cycles 10–11, 70% of which do not have introns and encode small proteins [6]. This is believed to be from selection for efficient transcription during the fast replication cycles in early embryonic development. Other studies in Drosophila have supported this idea [19], [20], [21]. We investigated *Ae. aegypti* EZG intron content by comparing them to genes expressed at 4–8 hr, 8–12 hr, and to all *Ae. aegypti* genes from the VectorBase AaegL1.1 database (Figure 2). As described, the EZG group contains those genes having transcripts at 2–4 hr that were not present at 0–2 hr. The 4–8 and 8–12 hr groups comprise genes with transcripts first showing presence at 4–8 hr and 8–12 hr, respectively, and are defined by the same criteria using DEGseq (P<0.001) (see File S3 for transcript IDs for 2–4, 4–8, 8–12 hr groups). Cellularization of the blastoderm in *Ae. aegypti* is reported to occur from 8–10 hr [7], so many of the genes first expressed in our 8–12 hr group can be considered post-cellular blastoderm. Only 10% of *Ae. aegypti* genes are without introns compared to 33% of the EZGs, and this difference is highly significant (P<0.0001). The difference between the 4–8 hr genes and all genes was also highly significant (P<0.0001). We observed a similar trend with respect to the mean number of introns per gene, EZGs having 1.78 introns per gene compared to 3.04 for all genes (not shown). Therefore we find evidence of selection against intron presence in EZGs.

### Discovery of a motif involved in early zygotic genome transcription

**Figure 3:**
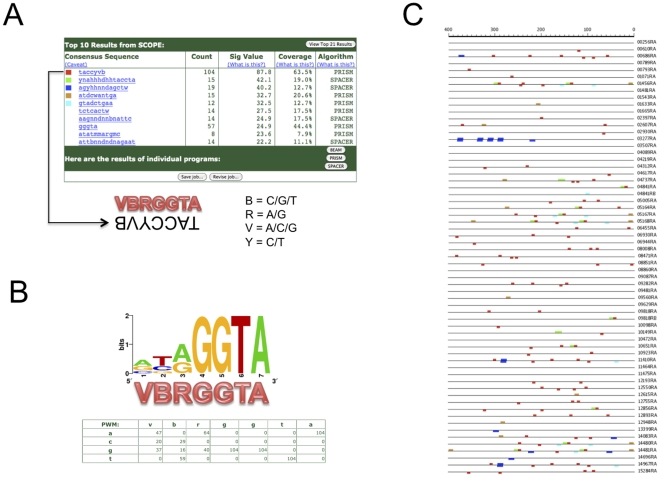
Discovery of a motif involved in early zygotic gene transcription. Analysis of 400 bp upstream sequence from 61 EZGs by SCOPE. A) Snapshot of SCOPE output showing the top 10 motifs identified. The top 5 are color-labeled and correspond to the motif locations in Figure 3C. Note that the highest-ranking motif TACCYVB is the complement of VBRGGTA. “Count” is the total motif occurrences on both strands, “Sig Value” is the negative log of the E value, “Coverage” is the % of sequences in the dataset that contain the motif, and “Algorithm” shows which of the three algorithms identified the motif. B) Sequence logos and PWM for VBRGGTA. C) Schematic from SCOPE output showing 400 bp upstream sequence and locations of 5 highest scoring motifs in Fig. 3A. Red blocks correspond to VBRGGTA. The gene IDs are shown to the right of each sequence with the prefix “AAEL0” omitted.

To identify a potential motif involved in early zygotic transcription, the upstream sequences of EZGs were analyzed using the web-based Suite for Computational Identification of Promoter Elements (SCOPE) [22], [23]. SCOPE has been shown to perform statistically better than many other motif-finding programs [23]. Our searches were performed with 200 to 2500 bp upstream sequence (see Materials and Methods). The motif with the highest significance score (87.8), VBRGGTA, was identified using 400 bp upstream sequence and had 63.5% coverage, meaning it was present at least once in 63.5% of the query sequences (Figure 3). Figure 3 shows the search results from using 400 bp upstream sequence. See File S4 for SCOPE output from searches using all upstream sequence lengths. In Figure 3A the complement sequence to VBRGGTA (TACCYVB) is shown in the SCOPE screenshot. In some cases, it appears that VBRGGTA is part of a much larger degenerate motif, the next highest scoring motif. In Fig. 3C the VBRGGTA (red blocks) overlap this other motif (green blocks). A similar motif VYRGGTA was found using 800 and 1000 bp upstream sequence, having significance scores of 86.4 and 83.2, and percent coverage in the query sequences of 74.6 and 79.4, respectively (File S4). Other GGTA-containing motifs similar to VBRGGTA were identified with lower significance but higher coverage. Some non-GGTA motifs were identified with higher significance scores, but did not have high coverage (25% or less). Most of these motifs were a part of repetitive sequences and were found in only one or a few sequences. Importantly, the VBRGGTA motif identified by SCOPE has both a high significance score and high coverage relative to all other motifs in the SCOPE output (File S4).

As a control, upstream sequences from 3 sets of 63 randomly chosen genes (File S3) were analyzed by SCOPE. An additional control was performed in a similar manner using datasets comprising transcripts first showing up significantly (P<0.001) at 4–8 hr and 8–12 hr. As before, 200–2500 bp upstream sequence was used as the input for SCOPE. No motif similar to VBRGGTA was identified in these datasets (not shown). Additionally, when VBRGGTA was used as a query to search these data sets using SCOPE, the significance score was negative for all searches performed (not shown).

### The *Ae. aegpyti* EZG and Drosophila TAGteam motifs may be homologous

Previous studies in Drosophila had identified the EZG TAGteam motif, CAGGTAG being the most overrepresented sequence [4]. The mosquito EZG motifs VBRGGTA/VYRGGTA (V = A/C/G, B = C/G/T, R = A/G, Y = C/T) resemble the TAGteam motif so we investigated the possibility that the mosquito and Drosophila motifs may be homologous. SCOPE was used to search the upstream sequences of two Drosopila EZG datasets from previous studies [4], [6] to find TAGteam-like motifs and generate position weight matrices (PWMs). Searches were performed in a similar manner as described above. SCOPE successfully identified TAGteam sites in both of these datasets, finding the degenerate motifs YAGGTAG and YAGGTA. It is important to note that for both of these TAGteam motifs identified by SCOPE, the Y in YAGGTAG/YAGGTA is comprised of 2.3 times C as T, making the motif consensus CAGGTAG or CAGGTA, matching the previously determined most overrepresented TAGteam motif CAGGTAG. Interestingly, CAGGTA has been reported as the most overrepresented TAGteam motif in hotspots for developmental transcription factor binding sites [24].

In order to establish significance of alignment between the mosquito and Drosophila motifs, the STAMP program was used [25]. STAMP is a program where a query motif sequence or PWM can be used to either search various databases with cis-regulatory sequences, or be aligned with a user-defined motif or PWM subject. The Drosophila TAGteam motifs are not in any of the databases currently available by STAMP. Therefore, the mosquito PWM for VBRGGTA was aligned with the two Drosophila motif PWMs identified from our SCOPE searches (Table 1). The mosquito motif was also aligned with the three previously reported TAGteam motifs [4]. These results suggest that the mosquito EZG and Drosophila TAGteam motifs may be homologous.

**Table 1:** Comparison of the *Ae. aegypti* EZG motif with Drosophila TAGteam motifs using STAMP.

Target Sequence	E value
TAGteam TAGGTAG^1^	3.60 e-7
YAGGTA PWM^2^	6.38 e-7
YAGGTAG PWM^3^	6.73 e-6
TAGteam CAGGTAG^1^	1.09 e-4
TAGteam CAGGCAG^1^	5.47 e-2

Notes:

1 TAGteam sites from [4].

2 Motif identified with SCOPE using previously published data [6].

3 Motif identified with SCOPE using previously published data [4].

PWM, Position Weight Matrix.

### Motifs in a 38 bp fragment of the kinesin light chain gene upstream sequence are necessary for early zygotic gene transcription

We reported the discovery of an early zygotic kinesin light chain gene *KLC2.1* and its paralog *KLC2.2* that are expressed exclusively in the early embryo (Biedler and Tu 2010). *KLC2.1* and *KLC2.2* are 12^th^ and 13^th^ in our list of most highly expressed EZGs (see File S1). We performed 5′RACE and identified the transcription start site (TSS) starting from the “T” in the last base of the arthropod initiator sequence “TCAGT” [26], resulting in a 39 bp 5′UTR. Counting from the first T, a “TATA” sequence is found at −30 from the TSS. We identified 5 VBRGGTA motifs in the 2500 bp upstream sequence from the start codon and all are concentrated within 265 bp upstream of the *KLC2.1* TSS. The slightly less degenerate VYRGGTA motif overlaps 4 of the 5 VBRGGTA motifs, including the two proximal sites investigated below. The 300 bp upstream sequences of *KLC2.1* and *KLC2.2* have 94% nt identity and the VBRGGTA and VYRGGTA sites are conserved.

**Figure 4:**
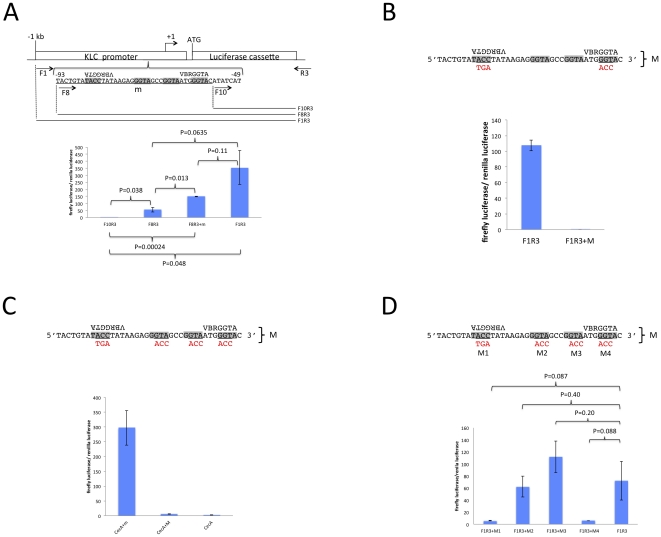
A 38 bp region of the *KLC2.1* promoter containing two VBRGGTA motifs is necessary and sufficient to direct early zygotic transcription. A) Duplication of the 38 bp fragment that contains the motifs enhances early zygotic transcriptional activity. Schematic shows constructs and upstream sequences tested with positions given relative to the TSS (+1). The 38 bp fragment containing 2 VBRGGTA (4 GGTA/TACC motifs shaded in gray) is underlined and labeled “m”. Primer locations used for PCR are depicted by arrows and labeled lines show sequence regions used for luciferase assay. Construct F10R3 doesn't contain the 38 bp fragment. F8R3 contains the 38 bp fragment. F8R3+m has a duplication of the 38 bp fragment. F1R3 is a positive control that contains the full 1 kb upstream/promoter sequence and is used hereafter as a positive control. B) Simultaneous mutations M1 and M4 (F1R3+M) in the 38 bp fragment obliterates activity. C) Addition of the 38 bp fragment confers early zygotic activity to a heterologous *AgCecA* promoter from a divergent mosquito species. CecA refers to the construct with the unmodified *AgCecA* promoter. CecA+m refers to the CecA construct with the addition of the 38 bp fragment. CecA+M refers to the CecA construct that contains the 38 bp fragment with mutations in all 4 positions indicated as in Figure 4B. A one-tailed T-test revealed significant differences between both CecA+m/CecA+M (*1, P = 0.019) and CecA+m/CecA (*2, P = 0.018). D) Mutational analysis of each individual motif in the 38 bp fragment. Each mutant is labeled M1–M4 and mutated bases are indicated in red. In all panels the positions of the VBRGGTA motif are indicated, and the ratio of firefly luciferase/renilla luciferase represents the mean of triplicate injections +/− the standard error.

We investigated the 1 kb upstream sequence for early zygotic promoter activity by embryo injection and luciferase reporter assays. Due to the high similarity of upstream sequences for *KLC2.1* and *KLC2.1* mentioned above and their identical expression profiles by RT-PCR [16], we assumed promoter activity of these genes would likely be similar and therefore decided to focus on *KLC2.1*. Initial experiments with 12 deletion series constructs of this 1 kb sequence identified the most significant loss of activity occurring when a 38 bp fragment containing 2 VBRGGTA motifs was removed (not shown). These 2 VBRGGTA motifs are the closest to the TSS, within 86 bp (VYRGGTA overlaps these 2 VBRGGTA motifs). Experiments focusing on this 38 bp fragment are presented here (Figure 4). When only the upstream sequence that is 3′ of the 38 bp fragment is included (the region 3′ relative to the second VBRGGTA, indicated by F10R3), activity is obliterated (Figure 4A). When the 38 bp upstream fragment is included (indicated by F8R3), activity is obtained. A duplication of the 38 bp fragment (F8R3+m) increases activity about 3-fold relative to F8R3. When the full 1 kb native upstream sequence (F1R3) is used, higher activity is observed compared to F8R3 suggesting the presence of influential sequences upstream of the 38 bp fragment. These results indicate that the 38 bp fragment containing the 2 VBRGGTA sequences is important and potentially sufficient to confer early zygotic transcription.

We had initially suspected the short “GGTA” sequence to be part of a motif involved in early zygotic transcription as it was part of larger degenerate motifs identified by SCOPE. To determine specifically which of the motifs are responsible for activity in the 38 bp fragment, mutant constructs were designed where each of the 4 GGTA motifs were altered, two of which overlap VBRGGTA (Figure 4B). Activity was obliterated when motifs 1 and 4 were simultaneously mutated. In these experiments, the full 1 kb upstream sequence was used (indicated by F1R3). These results support VBRGGTA as a motif being involved in EZG transcription.

To test whether the 38 bp fragment could confer early zygotic activity to a heterologous non-EZG promoter from a divergent mosquito species, this sequence was inserted in the *An. gambiae* cecropin A (*AgCecA*) promoter [27] (Figure 4C). To show that this activity was indeed from the motif sequences in the 38 bp insert, a mutant of the same construct was designed where all GGTAs were altered. The resulting activity was virtually extinguished and similar to the activity obtained from the control having only the *AgCecA* upstream sequence. An earlier experiment that tested individual mutants of each of the 4 GGTA motifs for activity showed the greatest reduction in activity when the 1^st^ and 4^th^ GGTAs were mutated (Figure 4D). Altogether, these results implicate the VBRGGTA motif being involved in early zygotic transcriptional activity and the 38 bp fragment to be sufficient to confer EZG transcriptional activity to a non-EZG promoter.

## Discussion

### *Ae. aegypti* EZGs

We have presented the first genome-wide study of EZGs in a mosquito species and have identified the EZGs of *Ae. aegypti*. We emphasize that evidence for 1,529 EZG transcripts without maternal contribution was detected from a total of 16,789 annotated transcripts. From the 1,529 transcripts, 63 (P<0.001) and 143 (P<0.05) transcripts had a significant increase in expression at 2–4 hr. Certainly some transcripts not having a significant change in expression according to DEGseq may indeed be biologically relevant. Additionally, many annotated transcripts have only one or a few hits in the transcriptome data so determining their significance is difficult. We must add that slight variations in temperature and embryo collection times could affect the RNA populations in the samples.

It is possible that some of the detected 1,529 transcripts that had low expression could result from background expression. However, many of these with very low relative expression at 2–4 hr have a trend of increasing expression in each of the 3 samples covering 2–12 hrs, and a significant change in expression between two or more samples after 4 hr (see File S1). Some are predicted homologs to known Drosophila genes with developmental roles. For example, an ortholog (AAEL008336) of *D. melanogaster snail* is found in the list of 1,529, and while it has only 1 read hit at 2–4 hr, it has 91 and 159 hits in the 4–8 hr and 8–12 hr samples, respectively.

It is interesting that many of the 61 EZGs do not have any clear homologs in non-mosquito species. Our preliminary analysis shows that many of the 61 EZGs are mosquito-specific. Approximately one fourth of the developmental genes identified in Drosophila listed in two studies [4], [28] have a predicted one-to-one ortholog in *Ae. aegypti* according to VectorBase but these orthologs are not expressed until 4–8 hr or 8–12 hr in *Ae. aegypti* (not shown). Drosophila development occurs at a much faster rate compared to *Ae. aegypti* and 2–4 hr in *Ae. aegypti* is relatively very early in Drosophila development which can partly explain this observation. Currently we are working on a detailed evolutionary analysis of these genes.

Varied expression profiles were observed from our data (see File S1) and it will be interesting to investigate the responsible regulatory mechanisms. Transcriptional activators as well as factors involved in transcript destabilization will likely be revealed. Some transcripts exhibiting a transient profile were present at only 2–4 hr or only in the 2–4 hr and 4–8 hr samples. Some transcripts appeared to have a bimodal profile being present at 2–4 hr and 8–12 hr, but not at 4–8 hr. In addition to the EZG group (transcripts first detected at 2–4 hr), there were transcripts first detected at either 4–8 hr or 8–12 hr. Further investigation will be needed to verify these expression profiles. We now have more transcriptome data sets for other developmental time points and tissues that will allow further analysis of gene expression in a broader context.

### A TAGteam-like motif involved in mosquito early zygotic gene activation

We have discovered a motif that is significantly overrepresented in the upstream sequences of *Ae. aegypti* EZGs. Our experiments show that a 38 bp fragment containing multiple motifs both enhances early zygotic transcription and is capable of conferring early zygotic transcription to a heterologous promoter in *Ae. aegypti* embryos. This activity presumably results from the binding of a positively-acting transcription factor(s). We cannot rule out the potential influence of sequence flanking the motifs in the 38 bp fragment.

Our findings are important as it is the second demonstration of such a motif in an insect species that is involved in early zygotic genome activation. It is interesting that we find evidence for homology between the mosquito EZG motif VBRGGTA and the Drosophila TAGteam motif. We used a common method (SCOPE) to identify high scoring and highly represented motifs in EZG datasets from these two divergent species. Importantly, the mosquito motif aligns against TAGteam motifs with considerably low E values (Table 1). Additional support for a GGTA-containing EZG motif comes from preliminary searches using SCOPE with upstream sequences from 6 highly expressed EZGs from *An. stephensi*, a very divergent mosquito species relative to *Ae. aegypti*. The top-scoring motif identified was VGGTAV and it is present in all sequences (not shown).

We can speculate that there may be an EZG transcriptional activator in mosquitoes that is homologous to the Drosophila Zelda. A gene tree from Ensembl (http://metazoa.ensembl.org/Drosophila_melanogaster/Info/Index) [29] shows that Drosophila Zelda, or viefaltig (FBgn0259789) has 8 predicted homologs in *Ae. aegypti*, one of which is a predicted one-to-one ortholog (AAEL008722) (File S7). One-to-one orthologs are also predicted for two other mosquito species, *C. quinquefasciatus* and *An. gambiae*. The gene tree reveals a complex evolutionary history for this gene family. Similarity in the amino acid alignment is primarily restricted to the zinc finger domains (not shown). Drosophila Zelda is transcribed maternally. However, AAEL008722 shows up first in the 4–8 hr sample (see File S1). Therefore, although it is possible that AAEL008722 functions as an EZG activator, it does not reason that it is responsible for activation of EZGs transcribed at 2–4 hr. We are currently working toward identifying the factor(s) that bind to the *Ae. aegypti* VBRGGTA motif.

### Identifying components for a *Medea* gene-drive system in mosquitoes

Engineering of a *Medea* gene-drive system requires spatiotemporal-specific promoters to drive expression of both the maternal toxin and the zygotic antidote [17]. Using high-throughput transcriptome sequencing of different embryonic time points has enabled us to efficiently identify a number of candidate genes to provide zygotic-specific promoters. In addition to the *KLC2.1* promoter, we have demonstrated zygotic activity for other promoters identified by this work using luciferase reporter assays after embryonic injection (see File S6). We are now working on transgenesis experiments to establish the *in vivo* zygotic-specific nature of these promoters.

## Materials and Methods

### Illumina transcriptome sequencing

Illumina transcriptome sequencing, mosquito rearing, embryo collection, RNA Isolation, RT-PCR, embryo injection, and luciferase assay were described previously [16]. *Ae. aegypti* transcriptome sequencing (RNA-Seq) was performed on 4 embryonic samples of 0–2, 2–4, 4–8, and 8–12 hr using the Illumina platform. To establish relative transcript level in each sample, 16,789 annotated transcript queries from the VectorBase AaegL1.1 database (http://vectorbase.org/) based on the *Ae. aegypti* genome [30] were used in Blast [31] vs. the transcriptome reads. The hit counts were normalized according to transcript length and total hit count per sample resulting in values expressed as reads per kilobase per million mappable reads by Blast. See File S1 for transcript hit count data. RNA-Seq data is accessible at NCBI GEO (http://www.ncbi.nlm.nih.gov/geo/) with accession GSE34480.

Our choice of 0–2 hr embryo as a time of transcriptional quiescence was based on previously published developmental facts for *Ae. aegypti* and Drosophila. In Drosophila, zygotic transcription was found to start with a group of 59 genes beginning at cycles 10–11 at 1.5 hr [6], and pole cells form at cycles 9–10 at about 1.2 hr. Transcription in Drosophila has only rarely been reported before pole cell formation [3]. In *Ae. aegypti*, pole cells form at about 3 hr [7]. Major developmental events between Drosophila and *Ae. aegypti* are generally well conserved but occurs at a faster rate in Drosophila [7], [8], [9]. Additionally, a near linear correspondence in development was found between *An. gambiae* and Drosophila [32]. Given this information and assuming general developmental consistency we reasoned that the 0–2 hr embryo would be transcriptionally silent. However, we cannot rule out the possibility of transcription before 2 hr.

### RT-PCR

RT-PCR was performed on RNA isolated from *Ae. aegypti* embryos at one-hour intervals from zero to seven hours, using 25 cycles for PCR. To test for genomic DNA contamination, primers designed for ribosomal protein S7 were used with samples prepared as others for reverse transcription but without reverse transcriptase, obtaining a negative result. Primers used to amplify the transcripts with IDs AAEL008851, AAEL001543, and AAEL008722 were forward primer GACGGATGGGATATCAATACC (Tm = 61.7) and reverse primer AACATTTAAGGAACGTGGCAC (Tm-62.6), forward primer ACCAATTCCACTAAGGACCAG (Tm = 61.7) and reverse primer GTTCAACTAAGCTTCGTTCTTCTAG (Tm = 60.5), forward primer AACTGCACTGCCTGTAACAAG (61.9) and reverse primer GAATCTACGGATGTGCTATCATATC (Tm = 61.4), respectively. PCR cycling parameters included an initial denaturation of 94 C for 3 m followed by 25 cycles of 94 C for 30 s, 59 C for 30 s, and 72 C for 40 s, and a final step of 72 C for 5 m. Expected product sizes were approximately 500 bp.

### Differential gene expression analysis

A DEGseq method in the DEGseq package [18] was used to determine the significant fold changes of transcripts between different time ranges. Among all available models in DEGseq including Fisher's exact test (FET), the method using likelihood ratio test (LTR), the MA-plot-based method with random sampling model (MARS) and the MA-plot-based method with technical replicates (MATR), we used MARS that is the primary model of DEGseq. The input data were the raw counts of mapped reads as recommended for the methods based on the random sampling model in the DEGseq study. We chose two different p-value thresholds, 0.001 and 0.05, to identify differentially expressed genes. (The results with p-value and q-value [33] thresholds are shown in File S1). Statistical analysis for differential gene expression was performed on the Illumina transcriptome data by the Virginia Bioinformatics Institute Data Analysis Core at Virginia Tech in Blacksburg, VA (https://www.vbi.vt.edu/dac/).

### *Ae. aegypti* EZG structure

Four data sets were used to compare the number of genes without introns. EZG, 4–8 hr, and 8–12 hr gene sets were selected as described above. The data set comprising all *Ae. aegypti* genes was obtained from VectorBase (vectorbase.org). Because genes may have multiple associated transcripts, genes were scored for presence/absence of introns according to their cognate transcripts. Transcripts were assigned as either having at least one intron or no introns, using predictions according to VectorBase annotations of the AaegL1.2 database. P-values were determined by the Chi-squared test.

### Bioinformatic identification of an early zygotic motif

The Suite for Computational Identification of Promoter Elements (SCOPE) is a parameter-free program designed to identify cis sequences in a set of co-regulated genes (http://genie.dartmouth.edu/scope/). SCOPE was used to identify potential motifs involved in EZG transcription. Twenty-five hundred bp upstream sequence relative to the TSS for each of the 61 EZG genes (63 transcripts) was retrieved from VectorBase (Vectorbase.org). Because of incomplete annotation in VectorBase, some retrieved upstream sequences start from the start codon and not from the TSS. Analysis was performed with the “fixed” search option and with *Ae. aegypti* as the target genome, using 2500, 1500, 1000, 800, 400, and 200 bp upstream sequence as these are all the options available by SCOPE. SCOPE output shows the motif along with the Count (total motif occurrences in the whole dataset), Sig Value (negative log of the expectation value), Coverage (the percentage of sequences in the query sequences that contain at least once motif occurrence), and Algorithm (which of the 3 algorithms used by SCOPE that identified the motif). Control motif searches were performed with 3 sets of 61 randomly chosen genes that were obtained using an in-house Perl script. As an additional control, the upstream sequences of genes that had transcripts showing first presence at 4–8 hr and 8–12 hr (P<0.001) were used as for motif search and analysis as described above. All control data set sequences were retrieved using Vectorbase and their transcript IDs can be found in File S3.

SCOPE was used to identify TAGteam EZG motifs in data sets from previous Drosophila studies [4], [6]. The gene IDs from these data sets were used to retrieve 2500 bp upstream sequence from the TSS using Biomart at Ensemble (ensemble.org) with the Drosophila database BDGP5.25. The highest scoring motifs found by SCOPE in these data sets were found using 400 and 2500 bp upstream sequence, respectively.

### Motif analysis with STAMP

The STAMP program (http://www.benoslab.pitt.edu/stamp/) was used to establish significance of alignments between mosquito and Drosophila EZG motifs. The PWMs generated by SCOPE for the most significant motifs identified in the *Ae. aegypti* and Drosophila [4], [6] EZG data sets were aligned. Additionally, the mosquito PWM was aligned with the 3 TAGteam motifs CAGGTAG, CAGGCAG and TAGGTAG [4]. See above for description of motif identification using SCOPE. The motif PWMs from these data sets can be found in File S5.

### Plasmid construction for motif analysis

Different regions of 1 kb promoter sequence from the EZG AaKLC2.1 (gene ID: AAEL011410) [16] linked to a firefly luciferase reporter cassette cloned from pGL3-basic plasmid (Promega) were inserted into the pGEM-T Easy vector (Promega). Deletion series from F1R3 to F12R3 were constructed by PCR (Figure 4A). A pGL3-basic firefly luciferase reporter plasmid (Promega) containing approximately 655 bp of the *AgCecA* promoter [27] was used to test whether the early zygotic motif could confer early zygotic expression. A 38 bp region containing the native or mutated GGTA/TACC motifs was synthesized and inserted into the *KLC2.1* F1R3/F8R3 and the pGL3-*AgCecA* plasmid.

### Embryonic injection and luciferase assay

Experimental and control plasmids were injected at a concentration ratio 5∶1, respectively.

Dual luciferase assays were performed at 5 hr post-oviposition using lysates from injected embryos to investigate the promoter of the previously described kinesin light chain gene AAEL011410 (Biedler and Tu, 2010). The *Renilla* luciferase reporter pRL-null (Promega) containing the *D. melanogaster* actin promoter was used as an injection control. P-values were calculated by a one-tailed, TypeII T-test.

## Supporting Information

File S1 Blast hit counts for *Ae. aegypti* transcripts, DEGseq output with p-values and q-values.
(XLSX)

File S2 Numbers of genes with and without introns.
(XLSX)

File S3 Transcript IDs for 2–4, 4–8, 8–12 hr groups (P<0.001) by DEGseq, and IDs for random sequence datasets.
(XLSX)

File S4 Tabulated summary of SCOPE output.
(XLSX)

File S5 PWMs used in STAMP.
(DOCX)

File S6 Luciferase assay results for other EZG promoters.
(PPTX)

File S7 Phylogeny of Zelda homologs from mosquito and Drosophila.
(PDF)
