# Supplementary material for: Identification of Early Zygotic Genes in the Yellow Fever Mosquito Aedes aegypti and Discovery of a Motif Involved in Early Zygotic Genome Activation
Source: PLoS One. 2012 Mar 23;7(3):e33933. doi: 10.1371/journal.pone.0033933 (PMC3311545; doi:10.1371/journal.pone.0033933)
Supplement: File S5 — PWMs used in STAMP. (DOCX) [file pone.0033933.s005.docx]

S5. Position Weight Matrices (PWM) used for motif analysis shown in Table 1. 100, 200, 400, 800, 1000, 1500, and 2500 bp upstream sequence was searched and the PWM was used from the search producing the most significant motif score for each dataset.

A) PWM generated by SCOPE using 400 bp upstream sequence from EZGs identified by DEGseq (P < 0.001).

>EZG_VBRGGTA_400

47 20 37 0

0 29 16 59

64 0 40 0

0 0 104 0

0 0 104 0

0 0 0 104

104 0 0 0

B) PWM generated by SCOPE using 400 bp upstream sequence from Drosophila [[1](#_ENREF_1)].

**>YAGGTA_PWM_Renzis_400**

0 100 0 43

143 0 0 0

0 0 143 0

0 0 143 0

0 0 0 143

143 0 0 0

C) ) PWM generated by SCOPE using 400 bp upstream sequence from Drosophila [[2](#_ENREF_2)].

>YAGGTAG_PWM_Bosch_2500

0 45 0 20

65 0 0 0

0 0 65 0

0 0 65 0

0 0 0 65

65 0 0 0

0 0 65 0

1. De Renzis S, Elemento O, Tavazoie S, Wieschaus EF (2007) Unmasking activation of the zygotic genome using chromosomal deletions in the Drosophila embryo. PLoS Biol 5: e117.

2. ten Bosch JR, Benavides JA, Cline TW (2006) The TAGteam DNA motif controls the timing of Drosophila pre-blastoderm transcription. Development 133: 1967-1977.
