# Supplementary material for: Identification of Early Zygotic Genes in the Yellow Fever Mosquito Aedes aegypti and Discovery of a Motif Involved in Early Zygotic Genome Activation
Source: PLoS One. 2012 Mar 23;7(3):e33933. doi: 10.1371/journal.pone.0033933 (PMC3311545; doi:10.1371/journal.pone.0033933)
Supplement: File S6 — Luciferase assay results for other EZG promoters. (PPTX) [file pone.0033933.s006.pptx]

## Slide 1
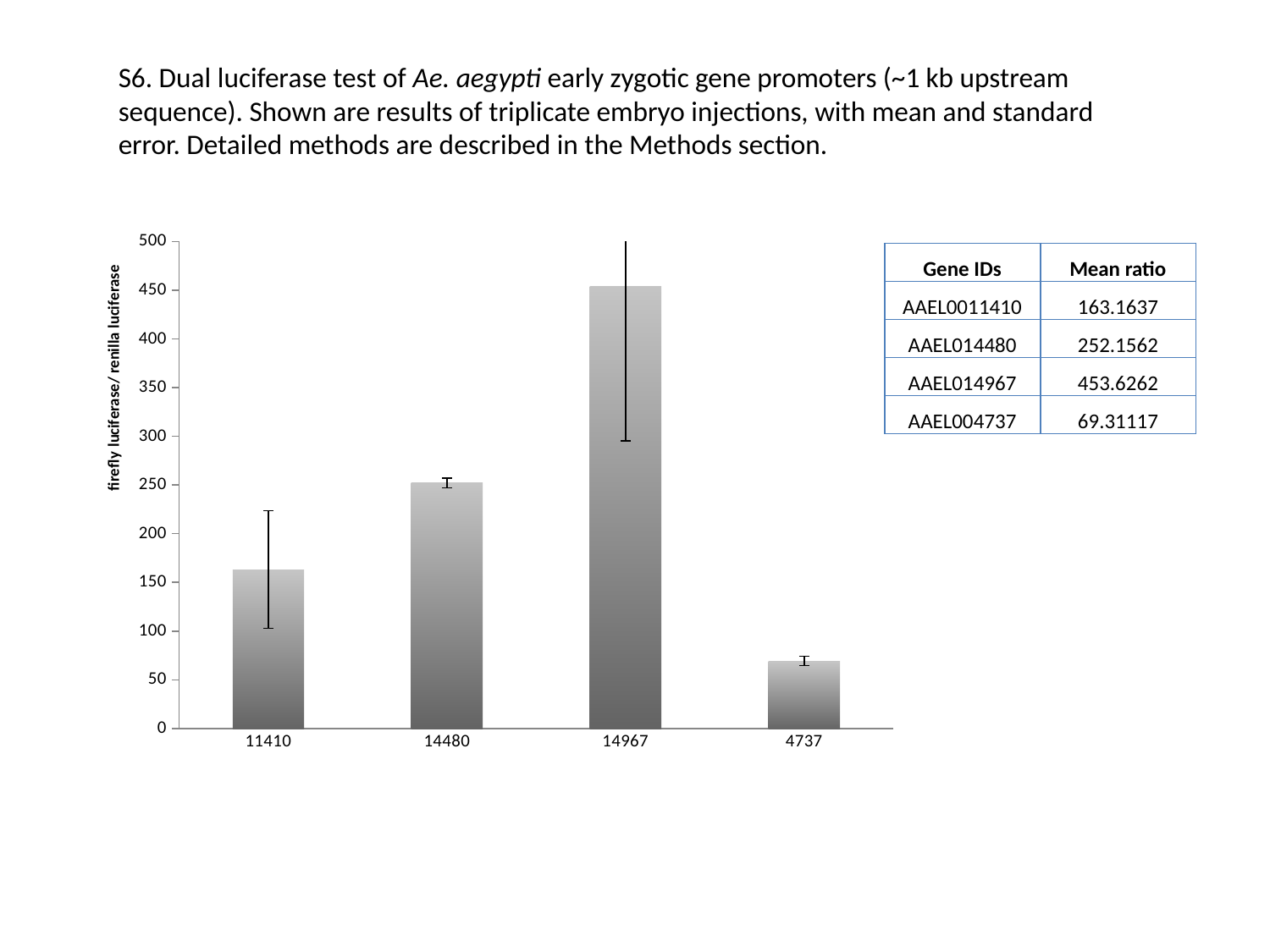

S6. Dual luciferase test of Ae. aegypti early zygotic gene promoters (~1 kb upstream sequence). Shown are results of triplicate embryo injections, with mean and standard error. Detailed methods are described in the Methods section.
### Chart
| Category | |
|---|---|
| 11410 | 163.163675 |
| 14480 | 252.1561666666666 |
| 14967 | 453.6261999999996 |
| 4737 | 69.31116666666667 || Gene IDs | Mean ratio |
| --- | --- |
| AAEL0011410 | 163.1637 |
| AAEL014480 | 252.1562 |
| AAEL014967 | 453.6262 |
| AAEL004737 | 69.31117 |
