# Supplementary material for: Identification of Early Zygotic Genes in the Yellow Fever Mosquito Aedes aegypti and Discovery of a Motif Involved in Early Zygotic Genome Activation
Source: PLoS One. 2012 Mar 23;7(3):e33933. doi: 10.1371/journal.pone.0033933 (PMC3311545; doi:10.1371/journal.pone.0033933)
Supplement: File S7 — Phylogeny of Zelda homologs from mosquito and Drosophila. (PDF) [file pone.0033933.s007.pdf]

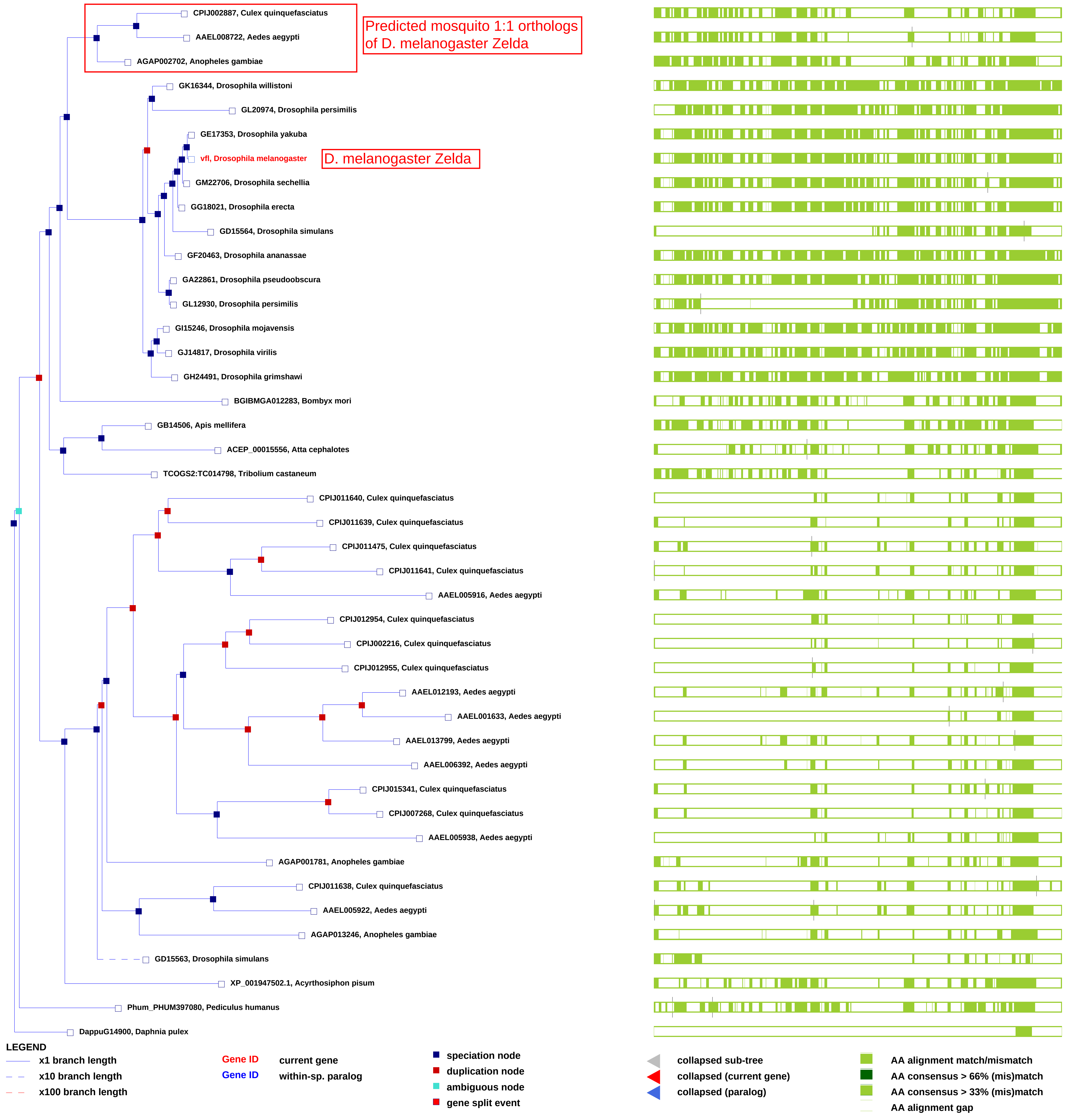

Predicted mosquito 1:1 orthologs of D. melanogaster Zelda
D. melanogaster Zelda
CPIJ002887, Culex quinquefasciatus
AAEL008722, Aedes aegypti
AGAP002702, Anopheles gambiae
GK16344, Drosophila willistoni
GL20974, Drosophila persimilis
GE17353, Drosophila yakuba
vfl, Drosophila melanogaster
GM22706, Drosophila sechellia
GG18021, Drosophila erecta
GD15564, Drosophila simulans
GF20463, Drosophila ananassae
GA22861, Drosophila pseudoobscura
GL12930, Drosophila persimilis
GI15246, Drosophila mojavensis
GJ14817, Drosophila virilis
GH24491, Drosophila grimshawi
BGIBMGA012283, Bombyx mori
GB14506, Apis mellifera
ACEP_00015556, Atta cephalotes
TCOGS2:TC014798, Tribolium castaneum
CPIJ011640, Culex quinquefasciatus
CPIJ011639, Culex quinquefasciatus
CPIJ011475, Culex quinquefasciatus
CPIJ011641, Culex quinquefasciatus
AAEL005916, Aedes aegypti
CPIJ012954, Culex quinquefasciatus
CPIJ002216, Culex quinquefasciatus
CPIJ012955, Culex quinquefasciatus
AAEL012193, Aedes aegypti
AAEL001633, Aedes aegypti
AAEL013799, Aedes aegypti
AAEL006392, Aedes aegypti
CPIJ015341, Culex quinquefasciatus
CPIJ007268, Culex quinquefasciatus
AAEL005938, Aedes aegypti
AGAP001781, Anopheles gambiae
CPIJ011638, Culex quinquefasciatus
AAEL005922, Aedes aegypti
AGAP013246, Anopheles gambiae
GD15563, Drosophila simulans
XP_001947502.1, Acyrthosiphon pisum
Phum_PHUM397080, Pediculus humanus
DappuG14900, Daphnia pulex
LEGEND
x1 branch length
x10 branch length
x100 branch length
Gene ID
current gene
Gene ID
within-sp. paralog
speciation node
duplication node
ambiguous node
gene split event
collapsed sub-tree
collapsed (current gene)
collapsed (paralog)
AA alignment match/mismatch
AA consensus > 66% (mis)match
AA consensus > 33% (mis)match
AA alignment gap
